# Exploring eye-hand coordination in central field loss with virtual reality

**DOI:** 10.21203/rs.3.rs-9839658/v1

**Published:** 2026-06-14

**Authors:** Jade Guénot, Preeti Verghese

**Affiliations:** Smith-Kettlewell Eye Research Institute; Smith-Kettlewell Eye Research Institute

## Abstract

Eye-hand coordination relies on accurate depth perception and gaze control, both of which are impaired in central field loss (CFL). Using an immersive VR paradigm, we examined how CFL affects gaze and hand control during a dynamic 3D butterfly-catching task. Seven patients with CFL and 13 controls performed three conditions: eye-tracking only (following the butterfly with gaze), hand-tracking (keeping the butterfly centered in a virtual net), and catching (capturing it). Patients performed all conditions binocularly and monocularly, while controls additionally performed them with a simulated scotoma. Binocular viewing led to faster catching times, and to more accurate gaze in both patients and controls compared to monocular viewing, with a greater binocular advantage in patients. Vergence analyses revealed that gaze followed the depth of the target more accurately during active catching in both groups, but only under binocular viewing, indicating that motor engagement refines depth estimates when binocular disparity is available. For hand-tracking, the ability to track the target in depth was particularly impacted in patients under monocular viewing, again suggesting a dependence on disparity information. Taken together, our VR studies reveal compensatory eye-hand strategies in CFL that support depth tracking under binocular viewing conditions that mimic real life.

## INTRODUCTION

Efficient interaction with moving objects in three-dimensions relies on precise coordination between visual perception and motor control. Eye-hand coordination supports a wide range of everyday behaviors, including reaching, grasping, tracking and intercepting dynamic targets, and depends on the integration of visual motion, depth information, and sensorimotor transformations ^1–3^. Accurate gaze control is a critical component of this process, as eye movements guide both perception and action by providing timely and spatially precise information about the target position.

Central field loss (CFL) caused by macular degeneration poses a real challenge to eye-hand coordination. The loss of foveal vision forces reliance on peripheral vision and an eccentric preferred retinal locus (PRL), which is associated with reduced spatial resolution due to the eccentricity from the fovea and impaired oculomotor control ^4–6^. Previous work has shown that CFL affects visually guided reaching and grasping, leading to increased movement variability, longer movement times, and reduced accuracy, particularly for depth-dependent actions ^7–10^. Depth perception deficits in CFL are further exacerbated under monocular viewing, as binocular cues become unavailable or unreliable ^11–13^.

Importantly, impairments in eye-hand coordination under visual loss, in particular when due to aged-related diseases such as macular degeneration, do not reflect motor deficits. Studies using simulated visual impairments have shown that occluding central or peripheral vision selectively alters coordination strategies, gaze allocation, and movement planning, often revealing compensatory behaviors rather than uniform degradation ^14–16^. These findings suggest that the sensorimotor system can flexibly adapt to degraded visual input by reweighting available cues.

Virtual reality (VR) provides a powerful framework to study such adaptations, as it enables controlled manipulation of visual inputs while allowing naturalistic, embodied interactions. VR-based studies have demonstrated that eye-hand coordination is sensitive to display modality, task instructions, depth cues, and the availability of haptic feedback ^17–20^. VR has also been increasingly used to assess and train eye-hand coordination in healthy individuals, athletes, and clinical populations, including stroke patients and individuals with visual impairment ^21–25^. However, much of this work has focused on discrete or predictable tasks, leaving open the question of how CFL affects eye-hand coordination during continuous interaction with unpredictably moving targets.

Moreover, although eye movements are often treated as leading hand actions, evidence suggests that hand movements can in turn influence gaze behavior. In some contexts, engaging the hand can stabilize gaze, reduce spatial uncertainty, or facilitate the detection of target motion, highlighting bidirectional coupling between eye and hand systems ^26,27^. Whether such eye-hand coupling can serve a compensatory role in CFL, particularly during dynamic 3D tasks, remains poorly understood.

In the present study, we used an immersive VR paradigm to investigate eye-hand coordination in individuals with CFL and normally sighted controls during interaction with an unpredictably moving target. Participants tracked and attempted to intercept a virtual butterfly whose speed and direction varied continuously, preventing anticipatory strategies. The task was performed under three conditions: gaze-only tracking, hand-based tracking with a virtual net, and target interception (catching), under monocular and binocular viewing, with controls additionally tested using gaze-contingent artificial scotomas. By analyzing continuous gaze and hand movements, gaze-target angular error, temporal coordination and task performance, we aimed to determine how central vision loss alters eye-hand coordination strategies, and whether engaging the hand can enhance gaze control and mitigate oculomotor deficits in CFL.

## METHODS

### PARTICIPANTS

Seven participants with macular degeneration (64–88 years old, four women) and 13 control participants (23–84 years old, 7 women) were included in this study. Control participants had normal or corrected-to-normal visual acuity, better than 0.1 logMAR in both eyes, and stereoacuity better than 100 arcsec. Their stereoacuity was measured using the Asteroid stereotest ^28^.

Six of the participants with MD had aged-related macular degeneration, and one had Stargardt’s disease. For participants with MD, coarse stereoacuity was measured with the Random Dot Stereo Butterfly Test. Their PRL eccentricity and stereo scotoma sizes were estimated using the Macular Integrity Assessment (MAIA; CenterVue, Padova, Italy) as described in ^29^. Table 1 reports the individual clinical characteristics of all participants with MD.

All experimental procedures were approved by the Institutional Review Board of the Smith-Kettlewell Eye Research Institute and followed the ethical standards of the Declaration of Helsinki. All participants gave informed written consent and received monetary compensation for their participation.

## MATERIALS

### Apparatus and Virtual Reality Setup

The experiment was conducted using an immersive virtual reality (VR) system. Participants wore an HTC Vive Pro Eye headset (Vive; HTC) equipped with an integrated eye-tracker. The head-mounted display (HMD) has two 3.5” OLED screens (1 per eye), each refreshing at 90 Hz with a resolution of 1440 × 1600 pixels (2880 × 1600 combined) giving the user a binocular field of view of ~ 72° wide and ~ 93° high, as measured by Aizenman and colleagues ^30^. Built-in eye tracking hardware collects gaze data with an accuracy of 0.5° – 1.1° within the central 20° field of view (according to official specifications: https://www.vive.com/ca/product/vive-pro-eye/specs/ and confirmed by Schuetz and Fiehler ^31^. We recorded eye-tracking data at 120 Hz sampling rate, head positions and rotations at 90 Hz (headset), and hands positions and rotations at 90 Hz (controllers). The VR environment was developed using Unity, and the experiment was implemented in Python using the Perception Toolbox for Virtual Reality (PTVR ^32^) which enabled precise control of stimulus properties and synchronization of eye, head, and hand movement data, as well as SRanipal software development kit (SDK; version 1.3.6.12), SteamVR (version 2.12.14). Eye, head, and controller data were recorded continuously throughout each trial.

### Virtual Environment and Stimulus

**Figure 1:**
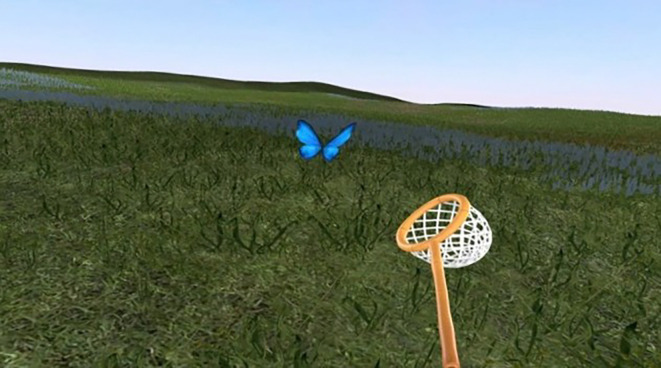
Screenshot of the virtual environment during the task. Participants were immersed in a field and viewed a butterfly, while holding a net. They had to either catch the butterfly, follow it with the net, or follow it with their gaze only.

Participants were immersed in a simple virtual environment with minimal visual clutter, a field with only grass on the ground. The target stimulus was a virtual blue butterfly with a wingspan of 8 cm (measured from the outermost tip of one wing to the outermost tip of the other, when the wings are fully spread) that moved within a three-dimensional workspace approximately 1 m × 1 m × 1 m in front of the participant (see Fig. 1). Participants held a physical hand controller that was rendered as a butterfly net (37 cm long, 15 cm net diameter at the opening part, and 14 cm deep for the white net part) in the virtual environment. The butterfly’s motion followed a pseudo-random trajectory, with frequent and unpredictable changes in direction and speed across all spatial axes. Average target speed was approximately 0.5 m/s, preventing participants from relying on predictive tracking strategies.

### Butterfly flight behavior

The butterfly was programmed to fly within an invisible virtual cubic area of 1 m^3^ positioned in front of the participant (in the virtual space). The area was defined to ensure that the butterfly remained within reach at all times, even at its furthest point, while preventing it from passing behind the headset or intersecting with the participant’s body. Additionally, a minimum flight altitude of 0.3 m was established to keep the butterfly visible above the ground and within the participant’s comfortable reaching range.

To produce a random flight pattern, random target positions within the cube were selected prior to the start of the butterfly movement at intervals between 1.5 and 3.5 seconds. Movement trajectories were then smoothed using Perlin noise functions that add naturalistic variations in all three spatial dimensions.

Position updates utilized Unity’s SmoothDamp function with a 0.3 second smoothing time constant, ensuring continuous trajectories without abrupt accelerations. The butterfly’s orientation was updated to align with its instantaneous velocity using linear interpolation (Slerp, creating realistic banking behavior during turns (e.g. the butterfly never flies on its back).

### Procedure

The experiment consisted of three task conditions presented in randomized blocks:

1. **Gaze-only tracking (gaze condition)**: Participants were instructed to track the moving butterfly (the target) using their gaze only. No hand movement was required. Each trial lasted 10 seconds.
2. **Hand-based tracking (net condition)**: Participants held a virtual net controlled by the handheld controller and were instructed to track the butterfly by keeping it as close as possible to the center of the net. Both eye and hand position were tracked. Trials lasted 10 seconds.
3. **Target interception (catching condition)**: Participants were instructed to catch the butterfly as quickly as possible by moving the net toward the target. Both eye and hand position were tracked. Each trial ended when the butterfly was successfully caught, with no time limit.

Each block comprised all three task conditions (10 trials per condition, 30 trials total), with the order of conditions randomized within each block. Participants were free to move their head during all conditions.

**Figure 2:**
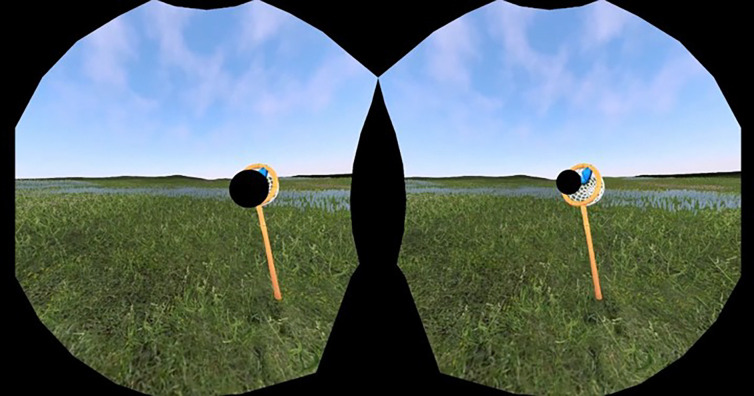
Screenshot of what the participant viewed with their left and right eye during the task, in the condition with scotomas. In the left eye, the scotoma measured 15° in diameter while the one in the right eye measured 10°.

Control participants completed the experiment under several viewing configurations, presented in randomized order: with or without artificial scotomas, with monocular (right eye only) or binocular vision. Artificial scotomas were gaze-contingent circular masks updated in real-time based on eye-tracking data at 120 Hz. In binocular-scotoma conditions, a 15° diameter scotoma was presented to the left eye and a 10° diameter scotoma to the right eye to approximate the asymmetric vision loss characteristic of macular degeneration (see Fig. 2). In monocular scotoma conditions, the same two gaze-contingent scotomas were used, while the butterfly and the net were rendered only to the right eye (which had the smaller scotoma, making it the “better” eye). This configuration simulated viewing with the better eye in participants with CFL, while maintaining scotoma presence. Participants with CFL completed the experiment binocularly and with monocular vision using their better-seeing eye and their worse eye (3 blocks total for patients, 4 blocks for control participants).

Each block began with a 5-point eye-tracker calibration. Participants were given the opportunity to rest between blocks to minimize fatigue. Each trial commenced with a 3-second countdown presented both visually (on-screen numerals) and aurally (beeps), during which participants prepared for tracking. At the end of the countdown, the butterfly appeared at a random location within the workspace in front of the participant. Participants first completed a brief familiarization session to become accustomed to the VR environment and task demands before beginning the experimental blocks.

### Data Acquisition and Preprocessing

Eye-tracking data included gaze direction vectors for each eye, which were combined with head orientation to compute gaze-in-world coordinates. Hand position and orientation were extracted from controller tracking data. All data streams were temporally aligned before analysis.

## DATA ANALYSIS

Analyses focused on task performance (time to capture the butterfly) and eye-hand coordination metrics derived from continuous gaze (eye+ head), and hand movements recordings. Three primary outcome measures were examined: (1) time to capture the butterfly in the catching condition, (2) gaze accuracy quantifying the angular error between gaze direction and the moving butterfly across all task conditions (gaze-only tracking, hand (net)-tracking, and catching), the vergence error and the vergence-distance correlation, and (3) hand tracking accuracy quantifying the distance error between the opening of the net and the moving butterfly. Additionally, we performed analysis on the gaze-target temporal coordination, measuring the temporal coordination between gaze and butterfly movements using cross-correlation analysis of position and velocity time series (see the Appendix). All analyses were conducted separately for patients with central vision loss and control participants, and compared across viewing conditions (binocular vs monocular). For controls, we additionally compared their performance with and without binocular scotomas. For patients, when performing monocular trials, we compared performance between viewing with the better-seeing eye versus worse eye.

### Time to capture

In the catching condition, performance was quantified as the time elapsed between trial onset and successful capture of the butterfly. Trial onset was defined as the first frame in which the butterfly appeared in front of the participant. Capture time was computed from system timestamps recorded at stimulus onset and at the moment of capture. A butterfly was considered successfully captured when the center of the butterfly entered the central capture region of the virtual net. No time limit was applied. Capture times were averaged across trials for each participant and conditions.

### Gaze and vergence error

To quantify gaze accuracy during butterfly tracking, we calculated three complementary metrics: the gaze angular error between the gaze direction and butterfly position, the vergence error between the participant’s estimated vergence distance and the butterfly’s physical distance, and the correlation between the vergence and the distance of the butterfly over time.

#### Gaze-target angular error.

Gaze direction was computed by combining eye vectors with head orientation to obtain gaze-in-world direction. Target direction was defined as the vector from the participanťs head position to the instantaneous position of the butterfly. The angular error was then computed as the 3D angle between the gaze-in-world vector and the target direction vector using the dot product of their normalized directions.

We transformed both butterfly position and the intersection of the binocular gaze vectors point into angular coordinates relative to the head's orientation, yielding azimuth (horizontal angle: 0° = straight ahead, positive = right) and elevation (vertical angle: 0° = horizontal plane, positive = up). Eye tracking data were filtered prior to analysis to remove blinks (eye openness < 0.1), invalid gaze samples (as flagged by the eye tracker's validity indicator), and gaze intersections with a distance greater than 10 meters from the participant’s head. To minimize noise from tracking artifacts, we applied a 5-sample median filter to the angular error time series and removed extreme outliers (> 90°). Data were acquired at 120 Hz for the eye tracker, and 90 Hz for head and butterfly, and spatially aligned across butterfly position, eye tracking, and head tracking streams using timestamp-based nearest-neighbor matching. Angular error values were summarized using the median angular error per trial to reduce sensitivity to outliers, then averaged across trials for each participant, condition, and block type.

#### Vergence error.

To assess depth-plane accuracy, we computed the vergence distance (i.e. the estimated depth at which the participanťs eyes converged) and compared it to the instantaneous distance of the butterfly from the participant’s head. The vergence point was estimated as the midpoint of the shortest segment connecting the left and right gaze rays, computed in local headset space. Vergence distance was defined as the Euclidean distance from the eye center (midpoint of the two eye origins) to the vergence point. Frames were excluded when the two gaze rays did not reliably converge, when the minimum distance between the two rays exceeded 0.05 m, or when vergence distance exceeded 10 meters. Blinks and invalid gaze samples were excluded using the same criteria as for angular error.

Vergence error was computed frame-by-frame as the signed difference between the reciprocal of vergence distance and the reciprocal of butterfly distance, expressed in diopters (positive values indicating over-convergence, i.e. vergence closer than the butterfly; negative values indicating under-convergence). Summary statistics (mean ± SD) were computed per trial, then averaged across trials per participant and condition.

#### Vergence-distance correlation.

Additionally, to quantify how well participants tracked the butterfly trajectory in depth over time, we computed the trial-by-trial correlation between the vergence distance time series and the butterfly distance time series. The resulting Pearson r value reflects the degree to which vergence distance co-varied with the butterfly distance within each trial, regardless of the absolute vergence level. Because Pearson r is bounded between − 1 and 1 and its sampling distribution is non-normal (particularly when values approach the bounds, as was the case in this dataset), r values were Fisher z-transformed prior to averaging and statistical analysis. Mean Fisher z values were computed per participant, condition, and block type.

Additional analyses were performed for gaze-target temporal coordination and are presented in the Appendix.

### Hand tracking: net to butterfly distance error

To quantify more precisely eye-hand coordination during butterfly tracking and catching, we computed the three-dimensional Euclidean distance between the center of the opening of the virtual net and the position of the butterfly. Since the eye-tracking block involved gaze recording only and no hand movement, net position data were not available for this condition. The catching block was also excluded from the present analysis as participants adopted highly variable strategies (i.e., some of them gradually tracked the butterfly before accelerating to catch it, others kept the net stationary and moved it at the last moment) and controls in particular often caught the butterfly in under a second, resulting in too few data points for a meaningful characterization of net-to-butterfly distance. This analysis therefore focused exclusively on the net-tracking block, in which hand movement was continuous and consistent across participants.

We computed signed directional errors along each world-space axis as the net - butterfly distance, yielding Δx (lateral), Δy (vertical), and Δz (depth). Positive values indicate that the net center was positioned to the right, above or behind the butterfly respectively, and negative values indicate the opposite. Net-butterfly distances were averaged across trials for each participant.

### Statistical Analysis

Statistical analyses were performed separately for each dependent variable: butterfly capture time, gaze-target angular error, vergence error, vergence-distance correlation, and net-butterfly 3D distance. Gaze-target temporal lag metrics (combined position lag and elevation lag, as well as velocity lag, and azimuth lag) are presented in the Appendix. Capture time was analyzed for the catching block only, and net-butterfly 3D distance for the net-tracking condition only. All other measures were analyzed across the three task conditions (eye-tracking, net-tracking, and catching).

#### Within-group analyses.

For controls, within-group effects were assessed using three-way repeated-measures ANOVAs with viewing condition (binocular vs monocular), scotoma condition (with scotoma vs without scotoma), and block type (eye-tracking, net-tracking, catching) as within-subjects factors. Capture time and net-butterfly 3D distance, which do not include a block type factor, were analyzed with two-way repeated-measures ANOVAs (viewing × scotoma).

Sphericity was assumed given the small number of levels per factor; when violated, Greenhouse-Geisser correction was applied. Significant interactions were decomposed using pairwise comparisons with Holm correction. Effect sizes are reported as partial eta-squared (η^2^p).

For patients, because of the absence of a “without scotoma” condition and the small sample size, we used parametric repeated-measures ANOVAs. Within-group effects were therefore assessed with Friedman tests, separately for (1) the effect of viewing condition (binocular scotoma vs monocular better eye vs monocular worse eye) within each block type, and (2) the effect of block type (eye-tracking vs net-tracking vs catching) within each viewing condition. Significant Friedman tests were followed by pairwise Wilcoxon signed-rank tests with Holm correction. Effect sizes are reported as Kendall's W (Friedman) and rank-biserial correlation r (Wilcoxon).

#### Between-group analyses.

Between-group comparisons were conducted using linear mixed models (LMMs) implemented in R with the lme4 package, with participant as a random intercept. The fixed-effects structure included group (patients vs controls), viewing condition (binocular vs monocular), block type (eye-tracking, net-tracking, catching), and all two- and three-way interactions. Only scotoma conditions present in both groups were included (binocular scotoma and monocular scotoma for controls; all three conditions for patients). Fixed effects were tested using Type III F-tests with Satterthwaite degrees of freedom (lmerTest package). Model comparison between the full interaction model and the additive model was performed using likelihood ratio tests (LRT). Estimated marginal means (EMMs) and pairwise contrasts were computed with the emmeans package, with Holm correction for multiple comparisons. Capture time and angular error were log-transformed prior to LMM analysis to meet normality assumptions; all other measures were analyzed on their original scale. Effect sizes for LMM contrasts are reported as Cohen's d computed from the t-statistic.

#### One-sample tests.

For vergence error, one-sample Wilcoxon tests against zero were performed (group × viewing × block type) to assess whether systematic biases in vergence distance were present; p-values were Holm-corrected across all cells within each group.

#### Normality.

Shapiro-Wilk tests were applied to residuals in the within-group ANOVA designs. Data failing the normality assumption (p < .05) are noted in the results; given the robustness of ANOVA to moderate violations and the consistency of results with non-parametric alternatives where tested, parametric results are reported throughout.

#### Multiple comparison correction.

Holm-Bonferroni correction was applied within each set of pairwise comparisons following a significant test, and within each set of one-sample tests. Correction was not applied across independent analysis. Statistical significance was set at α = .05 (two-tailed).

## RESULTS

We analyzed the participants’ performances across several behavioral measures. We first looked at the time to capture the butterfly, then we focused on the analysis of gaze and hand data to understand the reasons for the differences between the CFL and control groups. To do so, we analyzed the spatial accuracy of the visual tracking by looking at the gaze-target angular error, the vergence error and the vergence-distance correlation. Note that the gaze-target temporal coordination was also explored and is presented in the Appendix. We then computed the spatial accuracy of hand tracking by analyzing the net-butterfly 3D distance in both groups.

Each measure was examined across three task conditions (eye-tracking only, net tracking, and catching; with the exception of the net-butterfly 3D-distance), and across viewing configuration (binocular vs monocular, with vs without artificial scotoma for controls, and binocular vs monocular better eye vs monocular worse eye for patients). Statistical analyses consisted of within-group analyses examining how viewing configurations and task demands affected each group separately, between-group analyses comparing patients to controls overall and within specific viewing conditions, and focused analyses testing our primary hypothesis that hand involvement would differentially modulate gaze control in patients versus controls. Below we report results separately for each behavioral measure.

## TIME TO CAPTURE

### Within-group analyses.

**Figure 3:**
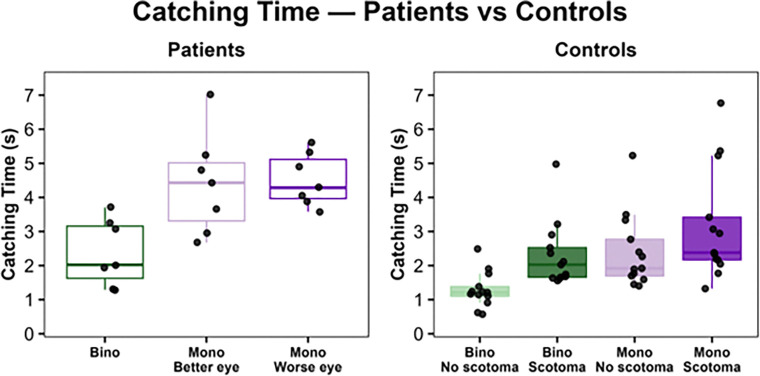
Catching time in seconds for patients (left panel) and controls (right panel), for each condition. Black dots represent individual means for each participant. Each boxplot shows the median threshold (dark internal horizontal line) and the first and third quartile (top and bottom edges of the box).

For controls, a 2×2 repeated-measures ANOVA on log-transformed capture times revealed significant main effects of both viewing condition (F(1,12) = 27.1, p < .001, η^2^p = .69) and scotoma condition (F(1,12) = 34.2, p < .001, η^2^p = .74), with no significant interaction (F(1,12) = 3.05, p = .106, η^2^p = .20; see Fig. 3 right panel). Capture times (back transformed) were faster in binocular viewing (M = 1.36 sec) compared to monocular viewing (M = 2.03 sec), and faster without artificial scotomas (M = 1.38 sec) than with scotomas (M = 2.00 sec). The absence of an interaction indicated that the cost of monocular viewing and the cost of scotoma presence were additive rather than synergistic.

For patients, a Friedman test revealed a significant effect of viewing condition (χ^2^(2) = 10.57, p = .005, Kendall's W = 0.76). Post-hoc Wilcoxon signed-rank tests with Holm correction showed that binocular viewing (median = 2.02 s, IQR = [1.63, 3.16]) was significantly faster than both monocular better-eye (median = 4.43 s, IQR = [3.31, 5.01]; W = 0, p = .047, r = .89) and monocular worse-eye conditions (median = 4.29 s, IQR = [3.97, 5.12]; W = 0, p = .047, r = .89; see Fig. 3 left panel). Notably, the statistic W = 0 indicates that all seven patients were faster in binocular than in either monocular condition, reflecting a highly consistent individual-level pattern. No significant difference was found between the two monocular conditions (W = 13, p = .938, r = .06), suggesting that the identity of the eye used did not modulate performance at group level. This could be due to our small sample size and large variability between patients, some of them having comparable vision in both eyes, while others had one eye much more affected by the disease.

### Between-group analyses.

A linear mixed model on log-transformed capture times, restricted to scotoma conditions present in both groups (binocular and monocular with scotoma for controls; all three conditions for patients), revealed a significant Group × Viewing interaction (F(1,447) = 6.39, p = .012) but no significant main effect of group (F(1,19.3) = 1.76, p = .200). This interaction indicates that the binocular advantage was more pronounced in patients than in controls. Estimated marginal means confirmed that the two groups performed comparably in binocular viewing (controls: 1.77 s [95% CI: 1.43–2.19]; patients: 1.82 s [95% CI: 1.35–2.43]), whereas patients were slower than controls in monocular viewing (patients: 3.29 s [95% CI: 2.51–4.31]; controls: 2.25 s [95% CI: 1.82–2.80]). It should be noted that patients were on average older than controls, which could at least partially explain their slower catching times.

**Figure 4:**
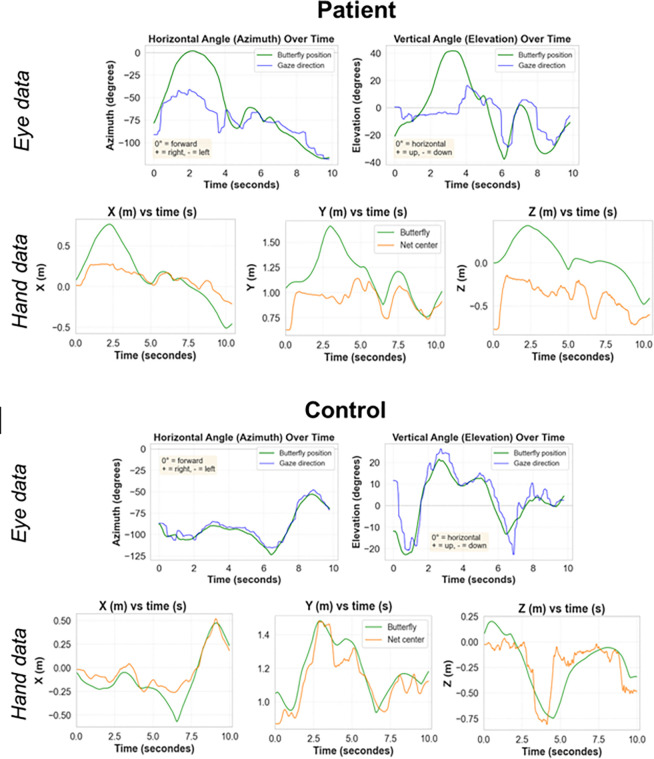
Examples of sample data recorded for one patient (MD7, top panel) and one control (bottom panel) during one trial of the net-tracking block. The first part of each panel shows in green the butterfly position over time for the horizontal (left) and vertical axis (right), and in blue the gaze data recorded. The second part of each panel shows the hand data collected during the same trial. The green line shows the position of the butterfly over time for the x, y and z axis, while the orange line represents the position of the center of the net opening. Note that vergence was also explored during our analysis (not shown on this figure).

Having established that binocular viewing and the presence of scotoma modulated the task performance, we next examined the effect of central vision loss on the ability to track the target. Specifically, we considered the tasks where observers were asked to just follow the target with their gaze, or to follow it with their gaze while manually tracking the target. Examples of sample data recorded for one patient and one trial are presented in the following figure (Fig. 4).

Both sets of sample data are from the net-tracking condition, and both participants are in their 80’s (MD7 who is 87 and a control participant who is 84 years old). As is apparent from the data, the control participant was able to track the butterfly more precisely both with their gaze and their hand. Our goal in the next Results sections is to quantify these differences between groups.

## GAZE–TARGET ANGULAR ACCURACY

We then examined whether the presence of a scotoma and viewing the target binocularly or monocularly affected the spatial accuracy of gaze during the butterfly tracking.

### Gaze angular error

#### Within-group analyses.

**Figure 5:**
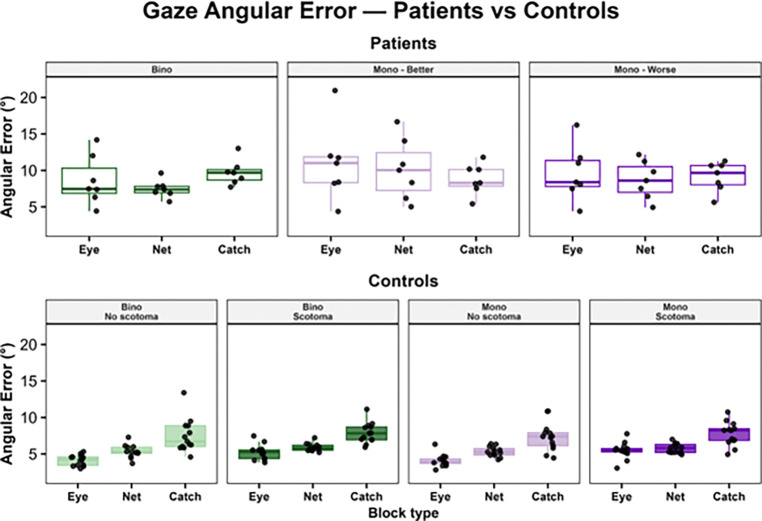
Angular errors in degrees for patients (top panel) and controls (bottom panel), for each viewing condition and each block type. Black dots represent individual means for each participant. Each boxplot shows the median threshold (dark internal horizontal line) and the first and third quartile (top and bottom edges of the box).

For controls, a 3×2×2 repeated-measures ANOVA on log-transformed median angular error revealed a significant main effect of the block type (F(2,22) = 53.1, p < .001, η^2^p = .82), with error increasing systematically from eye-tracking (mean: 4.04°) to net-tracking (5.18°) to catching (6.57°), all pairwise comparisons significant after Holm correction (all p < .001; see Fig. 5 bottom panel). A significant main effect of the scotoma condition was also observed (F(1,12) = 19.8, p = < .001, η^2^p = .62), with the artificial scotoma increasing angular error relative to the no-scotoma condition (means: 5.40° vs 4.96°). A significant scotoma × block type interaction (F(2,22) = 10.3, p = < .001, η^2^p = .46) indicated that the scotoma cost varied across tasks. No effect of viewing condition (binocular vs monocular) was found (F(1,12) = 0.058, p = .813).

For patients, Friedman tests revealed no significant effect of viewing condition on angular error for any of the three block types (eye-tracking: χ^2^(2) = 1.23, p = .540, W = .09; net-tracking: χ^2^(2) = 1.14, p = .565, W = .08; catching: χ^2^(2) = 2.00, p = .368, W = .14), indicating that gaze accuracy during butterfly tracking did not differ between binocular, better-eye monocular, and worse-eye monocular viewing (see Fig. 4 top panel). When examining the effect of the block type within each viewing condition, patients showed a significant task effect in binocular viewing only (χ^2^(2) = 6.00, p = .050, W = .43), driven by greater angular error in catching than in net-tracking (W = 0, p = .047, r = .89); no task effect was observed in either monocular condition (mono better eye: χ^2^(2) = 0.29, p = .867; mono worse eye: χ^2^(2) = 2.00, p = .368).

#### Between-group analyses.

Between-group comparisons using a linear mixed model on log-transformed angular error revealed a significant main effect of the group (F(1,20.3) = 18.5, p < .001), with patients showing consistently higher angular error than controls across all conditions. A significant group × viewing interaction (F(1,1390) = 6.08, p = .014) indicated that the binocular advantage for gaze accuracy was more pronounced in patients than in controls. Critically, a significant group × block type interaction (F(2,1390) = 13.0, p < .001) revealed that whereas controls showed a progressive increase in angular error from eye-tracking to catching (EMMs: 1.63 vs 1.93), patients maintained a relatively constant error level across tasks (2.08 vs 2.11). Pairwise contrasts confirmed that the group difference was largest for eye-tracking and net-tracking relative to catching (eye-tracking vs catching: estimate = − 0.254, p < .001; net-tracking vs catching: estimate = − 0.135, p = .008).

### Vergence error

Beyond the spatial accuracy of gaze direction, we also examined whether participants accurately tracked the butterfly in depth by analyzing the vergence error.

#### Within-group analyses.

**Figure 6:**
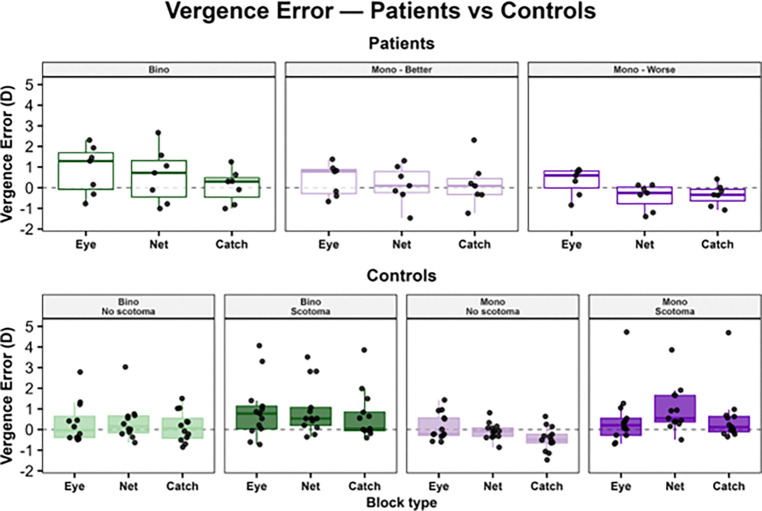
Vergence error in diopters for patients (top panel) and controls (bottom panel), for each viewing condition and each block type. The dashed line (0D) represents perfect depth-plane tracking, while a positive value indicates over-convergence, and a negative value under-convergence. Black dots represent individual means for each participant. Each boxplot shows the median threshold (dark internal horizontal line) and the first and third quartile (top and bottom edges of the box).

One-sample tests against zero (the value corresponding to perfect depth-plane tracking) revealed no significant deviations from zero in either group after Holm correction, indicating that participants converged at approximately the correct depth on average, but with considerable individual variability. For controls, a 3×2×2 repeated-measures ANOVA revealed a significant main effect of scotoma condition (F(1,10) = 12.2, p = .007, η^2^p = .50; see Fig. 6 bottom panel). The artificial scotoma induced systematic over-convergence relative to the butterfly's distance (scotoma: M = + 0.80 D; no scotoma: M = + 0.09 D). A main effect of the block type was also significant (F(2,20) = 4.42, p = .024, η^2^p = .29), as well as a significant three-way interaction between viewing condition, scotoma, and block type (F(2,20) = 4.64, p = .021, η^2^p = .30) suggested that the pattern of vergence error across tasks depended both on viewing mode and scotoma presence.

For patients, Friedman tests on the effect of viewing condition revealed no significant differences across block types (eye-tracking: χ^2^(2) = 5.85, p = .054, W = .42; net-tracking: χ^2^(2) = 3.71, p = .156, W = .27; catching: χ^2^(2) = 2.00, p = .368, W = .14), though the eye-tracking condition approached significance with a moderate-to-large effect size (see Fig. 5 top panel). When examining the effect of task within each condition, a pattern emerged for monocular viewing with the worse eye: vergence error differed significantly across tasks (χ^2^(2) = 10.57, p = .005, W = .76), with over-convergence during eye-tracking (median: +0.60 D) transitioning to under-convergence during both net-tracking (median: −0.25 D) and catching (median: −0.34 D; both pairwise comparisons: W = 28, p = .047, r = .89). No block type effect was observed for binocular viewing (χ^2^(2) = 2.00, p = .368) or monocular viewing with the better eye (χ^2^(2) = 0.00, p = 1.00).

#### Between-group analyses.

Between-group comparisons revealed significant main effects of viewing condition (F(1,1358) = 20.9, p < .001) and block type (F(2,1358) = 9.50, p < .001). A significant group × block type interaction (F(2,1358) = 6.95, p < .001) indicated that controls showed greater vergence error during net-tracking than during eye-tracking or catching (EMMs: +0.98 D vs + 0.73 D and + 0.60 D respectively), whereas patients maintained more uniform vergence error across tasks (net-tracking: +0.22 D; eye-tracking: +0.60 D; catching: +0.01 D). The group × viewing interaction approached significance (F(1,1358) = 3.39, p = .066), with a trend for greater vergence error in controls than patients during monocular viewing.

### Vergence-distance correlation

While vergence error captures the average deviation from the target’s depth plane, it does not capture how well participants tracked the continuous changes in the butterfly distance over time. Because participants with central vision loss experience a decreased ability to perceive depth ^33^, we also computed the trial-by-trial correlation between vergence distance and butterfly distance (Fisher z-transformed prior to analysis) as an additional index of depth tracking quality.

#### Within-group analyses.

**Figure 7:**
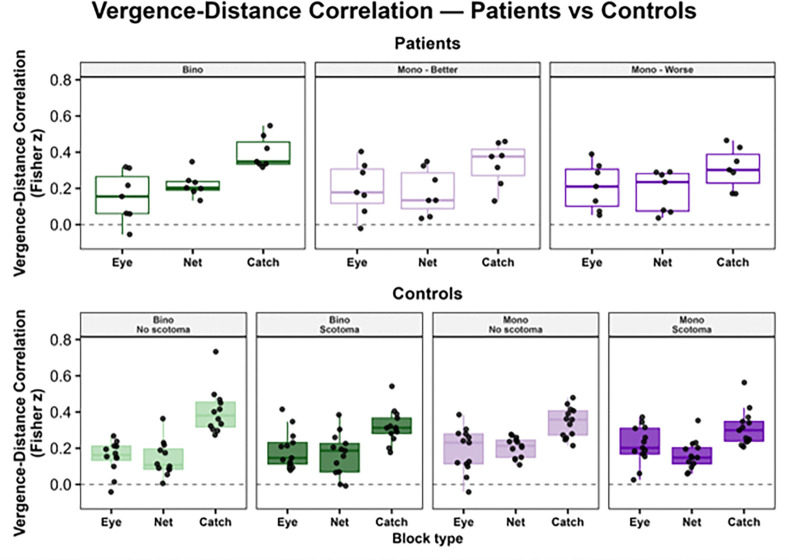
Vergence-distance correlation (Fisher Z) for patients (top panel) and controls (bottom panel), for each viewing condition and each block type. A higher value indicates a better depth tracking quality. Black dots represent individual means for each participant. Each boxplot shows the median threshold (dark internal horizontal line) and the first and third quartile (top and bottom edges of the box).

For controls, a 3×2×2 repeated-measures ANOVA revealed a dominant effect of the block type (F(2,20) = 41.4, p < .001, η^2^p = .79). The vergence-distance correlation was substantially higher during catching (Fisher z = 0.346) than during either eye-tracking (z = 0.189) or net-tracking (z = 0.166), with the latter two not differing from each other (eye-tracking vs catching: p < .001; net-tracking vs catching: p < .001; eye-tracking vs net-tracking: p = .099; see Fig. 7 bottom panel). A significant scotoma × block type interaction was also observed (F(2,20) = 4.20, p = .028, η^2^p = .28), indicating that the task-dependent modulation of vergence tracking quality differed between scotoma and no-scotoma conditions. No main effects of viewing condition or scotoma alone were significant.

For patients, Friedman tests on the effect of viewing condition revealed no significant differences for eye-tracking (χ^2^(2) = 2.15, p = .341, W = .15) or net-tracking (χ^2^(2) = 0.29, p = .867, W = .02). For the catching block, the result reached significance (χ^2^(2) = 6.00, p = .050, W = .43; see Fig. 6 top panel), though post-hoc Wilcoxon tests did not survive Holm correction (all p ≥ .141), despite large effect sizes for binocular versus monocular comparisons (r = .77 and r = .70). Regarding the effect of the block type, patients in binocular viewing showed a significant improvement in vergence-distance correlation from passive tracking to active catching (χ^2^(2) = 12.3, p = .002, W = .88). The correlation was higher during catching than during both eye-tracking (W = 0, p = .047, r = .89) and net-tracking (W = 0, p = .047, r = .89), with no difference between the net-tracking and the catching condition. Crucially, this task-dependent benefit was absent in both monocular conditions (mono better eye: χ^2^(2) = 2.00, p = .368; mono worse eye: χ^2^(2) = 3.71, p = .156), indicating that task demand improves depth tracking only when binocular disparity information is available.

#### Between-group analyses.

Between-group comparisons revealed no significant group differences in the vergence-distance correlation and no significant interactions involving group (all p ≥ .094; LRT comparing full vs additive model: χ^2^(7) = 7.76, p = .355). The dominant effect was task (F(2,1360) = 35.2, p < .001), with catching yielding higher vergence-distance correlations than passive tracking in both groups (controls catching EMM: z = 0.316; patients catching EMM: z = 0.361), confirming that the task demand benefit on depth tracking is preserved in patients with central field loss.

## HAND-TRACKING: NET-BUTTERFLY 3D DISTANCE

We then examined the accuracy of hand tracking by analyzing the net-butterfly 3D distance using data from the net-tracking block, in which hand movement was continuous and consistent across participants (see Methods, section net to butterfly distance error).

### Within-group analyses.

For controls, a 2-way repeated-measures ANOVA on mean 3D distance revealed a significant main effect of viewing condition (F(1,12) = 11.0, p = .006, η^2^p = .479), with no significant effect of scotoma condition (F(1,12) = 2.27, p = .158) and no interaction (F(1,12) = 0.022, p = .884). Distance was greater under monocular viewing (M = 0.518 m) than binocular viewing (M = 0.477 m; t(12) = 3.32, p = .006), indicating that the loss of binocular vision increased the net-to-butterfly distance during tracking. The presence of an artificial scotoma did not significantly modulate this distance.

For patients, a Friedman test revealed no significant effect of viewing condition on 3D distance (χ^2^(2) = 0.857, p = .651, W = .061), with median distances of 0.531 m (binocular), 0.459 m (monocular better eye), and 0.546 m (monocular worse eye). Distance to the butterfly thus did not differ significantly across viewing conditions in the patient group.

### Between-group analyses.

A linear mixed model revealed no significant main effect of group (F(1,19.8) = 0.470, p = .501) and no group × viewing interaction (F(1,449.9) = 0.858, p = .355), confirmed by a likelihood ratio test showing no improvement of the interaction model over the additive model (χ^2^(1) = 0.857, p = .355). A significant main effect of viewing condition was observed (F(1,449.9) = 6.14, p = .014), consistent with the within-group finding that monocular viewing increased net-to-butterfly distance in both groups (EMMs: controls bino = 0.491 m, controls mono = 0.534 m; patients bino = 0.537 m, patients mono = 0.557 m). These results indicate that patients and controls maintained comparable net-to-butterfly distances overall, with both groups showing a similar increase in distance under monocular viewing.

## DIRECTIONAL BIASES BY AXIS

Although no significant group differences were found for overall 3D distance, directional biases could exist anyway and be canceled out when combined. We therefore decomposed the net-to-butterfly error along each spatial axis separately to examine whether systematic biases existed, and whether these differed between groups or viewing conditions.

### Depth axis (Z).

**Figure 8:**
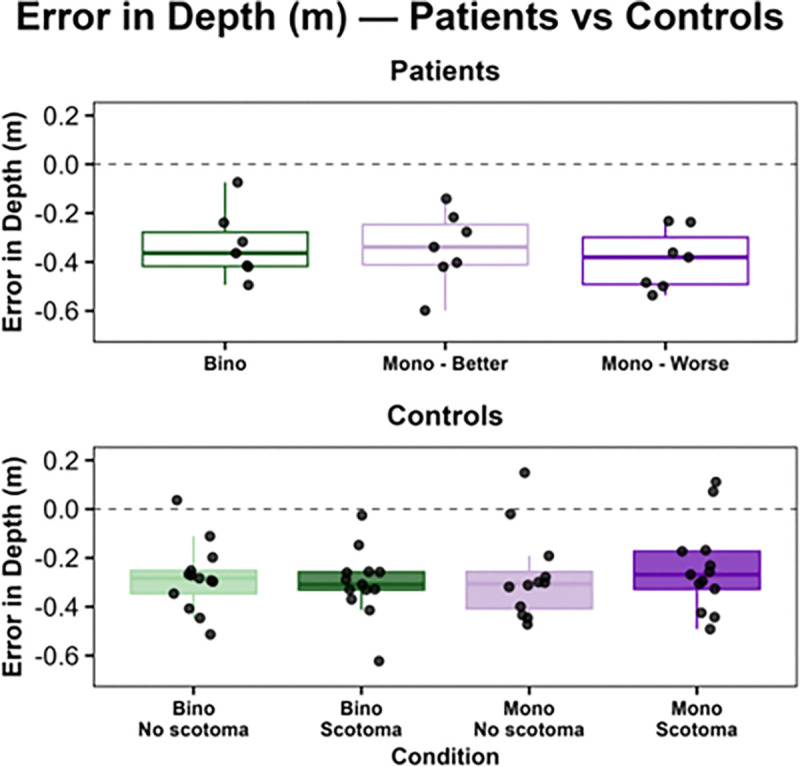
Error in depth (in meters). The error corresponds to the average distance between the butterfly and the center of the net for the depth (Z) axis, for patients (top panel) and controls (bottom panel) and for each viewing condition. Only the net-tracking block is considered for the analysis. A negative value indicates that participants undershoot the distance (i.e. the butterfly was farther away in depth compared to the net center). Black dots represent individual means for each participant. Each boxplot shows the median threshold (dark internal horizontal line) and the first and third quartile (top and bottom edges of the box).

The largest and most consistent bias was observed along the depth axis (see Fig. 8). One-sample tests revealed systematic negative values in both groups across all conditions (all p < .002 after Holm correction), indicating that participants consistently placed the net in front of the butterfly in depth, with mean biases ranging from − 0.246 to − 0.303 m for controls and from − 0.332 to − 0.390 m for patients. Note that the diameter of the net opening was 15 cm, which means that any value between − 7.5 cm and 7.5 cm indicates that the butterfly was inside the net.

For controls, a 2-way ANOVA revealed no significant effects of viewing condition, scotoma, or their interaction (all p > .20), indicating that this depth undershoot was stable across experimental manipulations. Similarly, patients showed no significant effect of viewing condition (χ^2^(2) = 0.857, p = .651, W = .061). Between-group analyses revealed a significant group × viewing interaction (F(1,449.7) = 5.63, p = .018), confirmed by a likelihood ratio test (χ^2^(1) = 5.59, p = .018). Controls showed a smaller depth undershoot in monocular than binocular viewing (EMMs: −0.246 m vs − 0.303 m), whereas patients showed the opposite pattern, with a larger undershoot in monocular viewing (− 0.366 m vs − 0.332 m). This crossover suggests that controls partially compensated for the loss of binocular disparity by adjusting their depth positioning, while patients did not.

### Lateral axis (X).

One-sample tests revealed systematic negative biases in both groups across all conditions (all p < .001 after Holm correction), indicating that the opening of the net was consistently placed to the left of the butterfly, which corresponds to having the butterfly inside the net. As a reminder, the white part of the net was 0.14 m deep, thus values below − 0.14 m indicate that the butterfly was outside of the net. Controls had mean biases ranging from − 0.089 to − 0.095 m and patients from − 0.138 to − 0.159 m, indicating that controls kept the butterfly perfectly inside the net, while the butterfly was sometimes slightly outside of it for patients.

Furthermore, for controls, a 2-way RM-ANOVA revealed no significant effects of viewing condition, scotoma, or their interaction (all p > .31). Similarly, for patients, a Friedman test revealed no significant effect of the viewing condition (χ^2^(2) = 0.286, p = .867, W = .020). Between-group analyses revealed a significant main effect of group (F(1,20.5) = 11.8, p = .003), with patients showing a consistently larger lateral bias than controls (EMMs averaged across conditions: −0.094 m for controls vs − 0.146 m). The group × viewing interaction approached but did not reach significance (F(1,450.7) = 3.66, p = .056), and the likelihood ratio test was similarly marginal (χ^2^(1) = 3.65, p = .056), suggesting a tendency for the lateral bias to be more strongly modulated by viewing condition in patients than in controls, though this effect was not conclusive.

### Vertical axis (Y).

Both groups showed systematic positive biases along the vertical axis (all one-sample tests p < .005 after Holm correction), indicating that the center of the net was consistently placed above the butterfly, with mean biases ranging from + 0.054 to + 0.069 m for controls and from + 0.089 to + 0.095 m for patients. As a reminder, the diameter of the opening net was 0.15 m, which means that any value between − 0.075 m and 0.075 m indicates that the butterfly was inside the net. Therefore, similarly to the lateral axis, control kept on average the butterfly inside the net, while the butterfly was slightly outside (under) of the net.

Moreover, for controls, a 2-way RM-ANOVA revealed a significant main effect of viewing condition (F(1,11) = 5.35, p = .041, η^2^p = .327), with a larger upward bias under monocular viewing (M = + 0.067 m) than binocular viewing (M = + 0.055 m; t(11) = 2.31, p = .041; both mean values still indicating that the butterfly was inside the net). Neither scotoma condition nor the interaction reached significance (all p > .18). For patients, a Friedman test revealed no significant effect of viewing condition (χ^2^(2) = 0.857, p = .651, W = .061). Between-group analyses revealed no significant main effect of group (F(1,20.0) = 3.24, p = .087), no significant main effect of viewing (F(1,450.2) = 1.66, p = .199), and no significant group × viewing interaction (F(1,450.2) = 1.93, p = .165), with the likelihood ratio test confirming no improvement of the interaction model (χ^2^(1) = 1.93, p = .165). Patients showed a numerically larger upward bias than controls across conditions (EMMs: patients bino = + 0.093 m, patients mono = + 0.092 m; controls bino = + 0.054 m, controls mono = + 0.069 m), but this difference did not reach statistical significance.

## DISCUSSION

The present study investigated how central field loss affects eye-hand coordination during interaction with a dynamically moving butterfly in a three-dimensional virtual environment. Using an immersive VR paradigm, we show that CFL alters both task performance and gaze control, and that active motor engagement during catching can partially improve depth tracking in participants with CFL, though without restoring the broader spatial accuracy of gaze guidance.

Both patients and controls benefited from binocular viewing when catching the butterfly, consistent with the well-established role of binocular vision in supporting depth perception and spatial accuracy ^11–13^. Controls showed significantly faster catching times in binocular compared to monocular viewing, while simulated scotomas further degraded performance, confirming that central vision contributes critically to efficient interception even in dynamic tasks. This is consistent with prior work showing that both real and simulated central vision loss impair visuomotor performance in reaching, grasping, and interception tasks ^7,8,10,14,16^.

Participants with CFL were overall slower than controls. This could be at least partially explained by the fact that participants with MD were on average older, and that motor latency declines with age ^34,35^. However, the disproportionate cost of monocular viewing seems to indicate that age does not entirely explain this group difference. All seven patients were faster in binocular than in either monocular condition, and performance was equally impaired regardless of whether the better or worse eye was used, suggesting that the binocular advantage in patients arises from disparity information. Notably, many long capture times (> 4 sec) in the monocular condition appeared to result from misestimation of the butterfly’s distance, suggesting impaired depth estimation rather than purely motor limitations, as the catching time was shorter binocularly. This is consistent with previous work showing that CFL is associated with increased uncertainty in depth judgments, particularly when binocular cues are reduced or unavailable ^9,10,29^.

Analyses of gaze accuracy revealed distinct patterns between patients and controls. In controls, gaze angular error increased progressively from eye-tracking, to net-tracking, to catching, suggesting a mild but consistent cost of adding manual task demands on gaze accuracy. This result is in line with dual-task interference, in which coordinating eye and hand movements may impose additional attentional or sensorimotor demands ^17,18^.

In contrast, patients with central field loss didn’t show such gradual increase in gaze angular error, which remained relatively constant across conditions. This pattern, confirmed by a significant group x block type interaction, indicates that patients, as a group, are less able to guide their gaze toward the target when task demand is low as in the eye-tracking condition. One possible explanation for showing persistently elevated error level is that the influence of motor demand on gaze guidance is harder to detect in patients because their baseline gaze error is substantially larger and more variable than in controls, even compared to controls performing under artificial scotomas. The relatively small task-dependent changes in gaze error that are clearly visible in controls may be obscured by the noisier gaze signal in patients, consistent with the instability of fixation and increased oculomotor variability associated with the use of a preferred retinal locus ^36^. A second interpretation is that controls, whose gaze tracking is near its accuracy limit during eye-tracking, show a measurable cost as soon as additional task demands are introduced, in line with evidence that increased cognitive or motor load degrades smooth pursuit and gaze accuracy even in normally sighted individuals ^37^. Patients, whose gaze tracking is already impaired because of their vision loss, may have less room to express such a graded response. Together, these interpretations suggest that the absence of task-dependent modulation in patients does not necessarily reflect a fundamental reorganization of gaze control, but may partly result from a ceiling on detectable change given their elevated baseline error. Regardless of the precise mechanism, the deficit cannot be attributed solely to the loss of central field input, but may also reflect long-term oculomotor adaptations specific to CFL.

Moreover, the analysis of vergence provided additional evidence for impaired depth tracking in participants with CFL. While participants in both groups converged at approximately the correct depth on average, considerable individual variability was observed. In controls, the presence of artificial scotomas induced systematic over-convergence, and vergence tracking quality (assessed via trial-by-trial vergence-distance correlations) was substantially higher during catching than during eye-tracking alone, indicating that task demand improves depth tracking in the presence of full binocular information. Critically, this motor demand benefit on vergence tracking was absent in monocular conditions, demonstrating that it depends on the availability of binocular disparity.

In patients, a comparable catching-related improvement in vergence-distance correlation was observed under binocular viewing only, and was similarly absent in monocular conditions. This convergence between groups suggests that the task benefit on depth tracking is preserved in CFL when binocular disparity is available, pointing to a specific contribution of signals from the hand in refining depth estimates during active catching. An additional finding was observed for patients viewing with their worse eye: vergence error shifted systematically across tasks, from over-convergence during passive gaze tracking to under-convergence during net-tracking and catching, suggesting that task demands may modulate depth estimates differently depending on residual visual input.

Gaze-target temporal coordination analyses, reported in the Appendix, further suggested that the motor-intention benefit on temporal gaze-target coupling may be absent or reduced in CFL, pointing to a qualitative reorganization of sensorimotor timing strategies.

Furthermore, analysis of hands movements revealed that both patients and controls maintained comparable overall net-to-butterfly distances, with both groups showing a similar increase in distance under monocular viewing during the net-tracking condition. This suggests that the ability to keep the hand in proximity to a moving target is broadly preserved in CFL, and that the loss of binocular vision affects hand guidance similarly in both groups. However, decomposing the tracking error along individual spatial axes revealed systematic directional biases that differed between groups in informative ways.

The most consistent hand-tracking bias across both groups was along the depth axis, with participants placing the net in front of the butterfly across all conditions. This depth undershoot likely reflects the inherent difficulty of estimating target distance in a dynamic 3D task, where depth cues are less reliable than lateral position cues. Critically, a significant group × viewing interaction revealed that controls partially compensated for the loss of binocular disparity by reducing their depth undershoot under monocular viewing, whereas patients showed the opposite pattern, with an increased undershoot in monocular conditions. This crossover suggests that normally sighted individuals can flexibly recalibrate their depth estimates when binocular cues are removed, potentially by upweighting monocular cues such as motion parallax or perspective^38^. In contrast, patients with CFL appear unable to deploy such compensatory recalibration, consistent with previous evidence that CFL is associated with impaired depth estimation and possibly reduced ability to integrate alternative depth cues ^10,29^ .

Despite an inability to correct the depth bias with visual cues to depth in monocular net-tracking conditions, our results support the idea that individuals with CFL adopt compensatory eye-hand coordination strategies during active dynamic tasks during binocular viewing. Rather than relying solely on gaze, patients appear to benefit from integrating hand-based information to partially improve depth tracking: active catching selectively enhanced vergence-distance correlation under binocular viewing conditions typical of everyday life, indicating that motor engagement can refine depth estimates when binocular disparity is available. Notably, this benefit was specific to the depth dimension: gaze angular error remained persistently elevated and constant across task conditions in participants with CFL, suggesting that hand involvement does not restore the broader spatial accuracy of gaze guidance. These findings align with broader evidence that sensorimotor integration is flexible and can be reweighted in the presence of sensory loss ^12,39^. In CFL, hand movements may partially substitute for degraded visual inputs to support depth tracking during active catching, suggesting a reorganization of the typical eye-hand hierarchy observed in normally sighted individuals. Yet this reorganization is incomplete as it does not fully compensate for the oculomotor limitations associated with CFL and does not restore the depth tracking quality or spatial calibration seen in controls.

The use of immersive VR was critical in revealing these effects, as it enabled precise measurement of eye, head, and hand movements during realistic interactions with a dynamic target. Traditional laboratory tasks may misestimate compensatory strategies that rely on active movement and embodied cues. From a clinical perspective, these results suggest that rehabilitation approaches for CFL could benefit from explicitly leveraging eye-hand coordination, for example by incorporating hand-guided tracking tasks or tool-based interactions. VR provides a promising platform to design, quantify, and adapt such interventions in a controlled yet ecologically valid way.

Finally, this study involved a limited number of patients, and the findings should therefore be interpreted as preliminary. Future work with larger samples will be needed to assess variability across individuals with CFL, including the role of preferred retinal locus characteristics and disease severity. Further studies could also examine whether similar compensatory mechanisms emerge in other dynamic tasks, such as obstacle avoidance or navigation.

## Supplementary Material

Supplementary Files

This is a list of supplementary files associated with this preprint. Click to download.

• GuenotVergheseScientificReportsAppendix.docx

## Figures and Tables

**Table 1:** Individual clinical characteristics of the participants with MD included in the study. *OS*: left eye; *OD*: right eye; *PRL eccentricity of DE*: preferred retinal locus of dominant eye.

Subject	Age	Sex	Diagnosis	Stereoacuity (arcsec)	Visual acuity (logMAR)		Dominant eye	PRL eccentricity of DE	Stereo scotoma size (max x and y)	Binocular scotoma size (max x and y)
					OS	OD				
MD1	73	F	AMD	160	0.36	0.34	OD	0.39°	Relative scotoma	No binocular scotoma
MD2	77	F	AMD	100	0.82	0.14	OD	1.67°	Relative scotoma	No binocular scotoma
MD3	83	M	AMD	100	0.92	0.9	OD	2.49°	2.25° x 1.79°	No binocular scotoma
MD4	88	M	AMD	200	1.2	1.3	OS	3.05°	14.92° x 10.7°	2.91° x 4.93°
MD5	85	F	AMD	200	0.74	0.36	OD	1.36°	11.41° x 13.81°	9.62° x 6.91°
MD6	64	M	Stargardt	1150	1.1	1.2	OD	5.24°	21.46° x 11.77°	15.18° x 9.67°
MD7	82	F	AMD	> 3000	0.86	0.96	OS	6.49°	21.28° x 20.11°	15.05° x 15.06°

## Data Availability

The data that support the findings of this study are available from the corresponding author upon reasonable request.

## References

[1] Henriques D. Y. P., Medendorp W. P., Khan A. Z. & Crawford J. D. Visuomotor transformations for eye-hand coordination. in Progress in Brain Research vol. 140 329–340 Elsevier, (2002). 12508600 10.1016/S0079-6123(02)40060-X

[2] Masia L., Casadio M., Sandini G. & Morasso P. Eye-Hand Coordination during Dynamic Visuomotor Rotations. PLoS ONE. 4, e7004 (2009). 19753120 10.1371/journal.pone.0007004 PMC2737429

[3] Zhang L., Guberman S. & Feldman A. G. Shifts in the eye-centered frame of reference may underlie saccades, visual perception, and eye-hand coordination. J. Neurophysiol. 128, 1025–1039 (2022). 36070246 10.1152/jn.00531.2021

[4] González E. G., Tarita-Nistor L., Mandelcorn E., Mandelcorn M. & Steinbach M. J. Mechanisms of Image Stabilization in Central Vision Loss: Smooth Pursuit. Optom. Vis. Sci. 95, 60–69 (2018). 29252901 10.1097/OPX.0000000000001161

[5] Shanidze N., Fusco G., Potapchuk E., Heinen S. & Verghese P. Smooth pursuit eye movements in patients with macular degeneration. J. Vis. 16, 1 (2016). 10.1167/16.3.1 PMC4748745 26830707

[6] Shanidze N., Heinen S. & Verghese P. Monocular and binocular smooth pursuit in central field loss. Vis. Res. 141, 181–190 (2017). 28057580 10.1016/j.visres.2016.12.013 PMC5502200

[7] Pardhan S., Gonzalez-Alvarez C. & Subramanian A. How does the presence and duration of central visual impairment affect reaching and grasping movements? Ophthalmic Physiol. Opt. 31, 233–239 (2011). 21410742 10.1111/j.1475-1313.2010.00819.x

[8] Sivak B. & MacKenzie C. L. Integration of visual information and motor output in reaching and grasping: The contributions of peripheral and central vision. Neuropsychologia 28, 1095–1116 (1990). 2267060 10.1016/0028-3932(90)90143-c

[9] Timberlake G. T., Omoscharka E., Quaney B. M., Grose S. A. & Maino J. H. Effect of Bilateral Macular Scotomas from Age-Related Macular Degeneration on Reach-to-Grasp Hand Movement. Invest. Ophthalmol. Vis. Sci. 52, 2540–2550 (2011). 21296817 10.1167/iovs.10-6062

[10] Verghese P., Tyson T. L., Ghahghaei S. & Fletcher D. C. Depth Perception and Grasp in Central Field Loss. Invest. Ophthalmol. Vis. Sci. 57, 1476–1487 (2016). 27031841 10.1167/iovs.15-18336 PMC4819556

[11] Loftus A., Servos P., Goodale M. A., Mendarozqueta N. & Mon-Williams M. When two eyes are better than one in prehension: monocular viewing and end-point variance. Exp Brain Res 158, (2004). 10.1007/s00221-004-1905-2 15164152

[12] Melmoth D. R., Finlay A. L., Morgan M. J. & Grant S. Grasping deficits and adaptations in adults with stereo vision losses. Invest. Ophthalmol. Vis. Sci. 50, 3711–3720 (2009). 19339741 10.1167/iovs.08-3229

[13] Verghese P. The utility of peripheral stereopsis. Front Neurosci 17, (2023). 10.3389/fnins.2023.1217993 PMC10545962 37795187

[14] Baker N. A. Effects of Central and Peripheral Vision Occlusion on Motor Performance during Hand Coordination Tasks. IISE Trans. Occup. Ergon. Hum. Factors. 5, 148–157 (2017).

[15] Dagnelie G. Hand-Eye Coordination in Virtual Reality under Simulated Ultra-Low Vision Conditions. Invest. Ophthalmol. Vis. Sci. 62, 3575–3575 (2021).

[16] Maiello G., Kwon M. & Bex P. J. Three-dimensional binocular eye–hand coordination in normal vision and with simulated visual impairment. Exp. Brain Res. 236, 691–709 (2018). 29299642 10.1007/s00221-017-5160-8 PMC6693328

[17] Batmaz A. U., De Mathelin M. & Dresp-Langley B. Seeing virtual while acting real: Visual display and strategy effects on the time and precision of eye-hand coordination. PLOS ONE. 12, e0183789 (2017). 28859092 10.1371/journal.pone.0183789 PMC5578485

[18] Cheng L. & Lin C. J. The effects of depth perception viewing on hand–eye coordination in virtual reality environments. J. Soc. Inf. Disp. 29, 801–817 (2021).

[19] Lavoie E., Hebert J. S. & Chapman C. Comparing eye–hand coordination between controller-mediated virtual reality, and a real-world object interaction task. J. Vis. 24, 9 (2024). 10.1167/jov.24.2.9 PMC10905649 38393742

[20] Lavoie E., Hebert J. S. & Chapman C. S. How a lack of haptic feedback affects eye-hand coordination and embodiment in virtual reality. Sci. Rep. 15, 25219 (2025). 40652089 10.1038/s41598-025-10319-0 PMC12255797

[21] Batmaz A. U., Sun X., Taskiran D. & Stuerzlinger W. Eye-Hand Coordination Training for Sports with Mid-air VR. in 26th ACM Symposium on Virtual Reality Software and Technology 1–10ACM, Virtual Event Canada, (2020). 10.1145/3385956.3418971

[22] Becker J. Augmented Reality Framework to Measure and Analyze Eye–Hand Coordination in Stroke Patients with Unilateral Neglect: Proof-of-Concept Study. JMIR XR Spat. Comput. 2, e70985–e70985 (2025). 42205919 10.2196/70985 PMC13202503

[23] Kartha A. Measuring visually guided motor performance in ultra low vision using virtual reality. Front. Neurosci. 17, 1251935 (2023). 38178831 10.3389/fnins.2023.1251935 PMC10765526

[24] Kartha A. Measuring visual information gathering in individuals with ultra low vision using virtual reality. Sci. Rep. 13, 3143 (2023). 36823360 10.1038/s41598-023-30249-z PMC9950080

[25] Mughrabi M. H. On The Effectiveness of Virtual Eye-Hand Coordination Training With Head Mounted Displays. in. IEEE Conference on Virtual Reality and 3D User Interfaces Abstracts and Workshops (VRW) 36–43 (IEEE, Shanghai, China, 2023). (2023). 10.1109/VRW58643.2023.00014

[26] Carrasco M. & Clady X. Exploiting eye–hand coordination to detect grasping movements. Image Vis. Comput. 30, 860–874 (2012).

[27] Pan Y., Sidenmark L. & Singh K. Head-EyeK: Head-Eye Coordination and Control Learned in Virtual Reality. IEEE Trans. Vis. Comput. Graph. 31, 9039–9051 (2025). 40663692 10.1109/TVCG.2025.3589333

[28] Vancleef K. ASTEROID: A New Clinical Stereotest on an Autostereo 3D Tablet. Transl Vis. Sci. Technol. 8, 25 (2019). 10.1167/tvst.8.1.25 PMC6396686 30834173

[29] Guénot J. & Verghese P. Cue combination for depth perception in macular degeneration: Motion parallax augments disparity. J. Vis. 26, 11 (2026). 10.1167/jov.26.1.11 PMC12849822 41569039

[30] Aizenman A. M. The Statistics of Eye Movements and Binocular Disparities during VR Gaming: Implications for Headset Design. ACM Trans. Graph. 42, 1–15 (2023). 10.1145/3549529 PMC10139447 37122317

[31] Schuetz I. & Fiehler K. Eye tracking in virtual reality: Vive pro eye spatial accuracy, precision, and calibration reliability. J Eye Mov. Res 15, (2022). 10.16910/jemr.15.3.3 PMC10136368 37125009

[32] Castet E. PTVR – A software in Python to make virtual reality experiments easier to build and more reproducible. J. Vis. 24, 19 (2024). 38652657 10.1167/jov.24.4.19 PMC11044846

[33] Cao K. Y. & Markowitz S. N. Residual stereopsis in age-related macular degeneration patients and its impact on vision-related abilities: A pilot study. J. Optom. 7, 100–105 (2014). 24766867 10.1016/j.optom.2013.12.003 PMC4009455

[34] Seidler R. D. Motor control and aging: Links to age-related brain structural, functional, and biochemical effects. Neurosci. Biobehav Rev. 34, 721–733 (2010). 19850077 10.1016/j.neubiorev.2009.10.005 PMC2838968

[35] Spirduso W. W. Reaction and Movement Time as a Function of age and Physical Activity Level. J. Gerontol. 30, 435–440 (1975). 1141674 10.1093/geronj/30.4.435

[36] Crossland M. D., Sims M., Galbraith R. F. & Rubin G. S. Evaluation of a new quantitative technique to assess the number and extent of preferred retinal loci in macular disease. Vis. Res. 44, 1537–1546 (2004). 15126063 10.1016/j.visres.2004.01.006

[37] Hutton S. B. & Tegally D. The effects of dividing attention on smooth pursuit eye tracking. Exp. Brain Res. 163, 306–313 (2005). 15654587 10.1007/s00221-004-2171-z

[38] Landy M. S., Maloney L. T., Johnston E. B. & Young M. Measurement and modeling of depth cue combination: in defense of weak fusion. Vis. Res. 35, 389–412 (1995). 7892735 10.1016/0042-6989(94)00176-m

[39] Ernst M. O. & Banks M. S. Humans integrate visual and haptic information in a statistically optimal fashion. Nature 415, 429–433 (2002). 11807554 10.1038/415429a

